# Glucagon Like Peptide-1 Promotes Adipocyte Differentiation via the Wnt4 Mediated Sequestering of Beta-Catenin

**DOI:** 10.1371/journal.pone.0160212

**Published:** 2016-08-09

**Authors:** Rui Liu, Na Li, Yi Lin, Mei Wang, Yongde Peng, Keidren Lewi, Qinghua Wang

**Affiliations:** 1 Department of Endocrinology, Huashan Hospital, Fudan University, Shanghai 200040, China; 2 Department of Endocrinology, Shanghai First People’s Hospital, Shanghai Jiao TongUniversity, Shanghai 200080, China; 3 Division of Endocrinology and Metabolism, Keenan Research Centre for Biomedical Science of St. Michael's Hospital, Departments of Physiology and Medicine, University of Toronto, Toronto, M5B 1W8, Canada; Western University, CANADA

## Abstract

Glucagon-like peptide-1 (GLP-1) plays a role in the regulation of adipogenesis; however, the precise underlying molecular mechanism has not been fully defined. Wnt was recently identified as an important regulator of adipogenesis. This study aimed to investigate the involvement of the Wnt signaling pathway in the effects of GLP-1 on adipocyte differentiation. 3T3-L1 cells were induced to differentiate. The changes in the expression levels of adipogenic transcription factors and Wnts and the phosphorylation level and subcellular localization of β-catenin were quantified after GLP-1 treatment. GLP-1 stimulated adipocyte differentiation and lipid accumulation, which were accompanied by the expression of adipocyte marker genes. The expression of Wnt4 was upregulated in the process of adipocyte differentiation, which was further enhanced by treatment with GLP-1. β-catenin, an important mediator of the Wnt pathway, was immediately dephosphorylated and translocated from cytoplasm to nucleus when differentiation was induced. In the presence of GLP-1, however, β-catenin was redirected to the cell plasma membrane leading to its decreased accumulation in the nucleus. Knockdown of Wnt4 blocked the effect of GLP-1 on the cellular localization of β-catenin and expression level of adipogenic transcription factors. Our findings showed that GLP-1 promoted adipogenesis through the modulation of the Wnt4/β-catenin signaling pathway, suggesting that the GLP-1-Wntβ-catenin system might be a new target for the treatment of metabolic disease.

## Introduction

Chronic nutrient overload causes an increase in adipose depots. This effect has often been associated with the development of type 2 diabetes, atherosclerosis, and hyperlipidemia [1;2]. The growth of adipose tissue involves cellular hypertrophy (cell size increase) and hyperplasia (cell number increase) [3]. It is believed that, in adult humans,the recruitment and proliferation of pre-adipocytes occur in addition to adipocyte hypertrophy [4;5]. In fact, during persistent positive caloric balance, if adipogenesis is impaired after initial adipocyte hypertrophy, then further adipocyte hypertrophy can result in adipocyte metabolic and immune abnormalities [6;7]. Therefore, adipogenesis is an important physiological process, and its function or dysfunction could prevent or promote metabolic disease [6;8].

The stimulation of adipogenesis consists of the sequential activation of a transcription factor cascade [9]. The transient induction of CCAAT/enhancer-binding protein-β (CEBPB) and -δ (CEBPD) directly induces expression of CEBP-α (CEBPA) and peroxisome proliferator-activated receptor-r (PPARG). Subsequently, many downstream target genes are activated, the expressions of which define the adipocytes, including lipoprotein lipase (LPL), adipocyte protein 2 (aP2) and adiponectin [9;10]. This process is regulated by the balance of local and endocrine factors, which either stimulate or inhibit the differentiation of pre-adipocytes into adipocytes [11]. Well-known factors that stimulate differentiation include glucocorticoid agonists, high concentrations of insulin, PPARG agonists and agents that elevate cAMP [12]. Factors that counteract these positive stimuli include wingless-type MMTV integration site family members (Wnts), tumor necrosis factor α (TNFα), transforming growth factorβ (TGFβ), epidermal growth factor, and prostaglandin F2A [11]. Among these factors, Wnt is believed to be an important physiological regulator of adipogenesis [13;14].

The Wnts are a family of secreted proteins that act through paracrine and autocrine mechanisms to regulate many aspects of cell fate and development [15]. The canonical Wnt signaling cascade converges on the transcription factor β-catenin. In the absence of Wnts, cytoplasmic β-catenin is recruited to a degradation complex nucleated by Axin and adenomatous polyposis coli (APC), facilitating its sequential phosphorylation by casein kinase I and glycogen synthase kinase-3β (GSK3β). This phosphorylation primes β-catenin for ubiquitination and proteasomal degradation. The binding of Wnt to frizzled (FZD) receptors and low-density lipoprotein-receptor-related protein-5 and -6 (LRP5/6) co-receptors leads to inactivation of the degradation complex, resulting in the hypophosphorylation of β-catenin and its translocation to the nucleus, where it binds to the lymphoid-enhancer-binding factor/T-cell-specific transcription factor (LEF/TCF) family of transcription factors and activates Wnt target genes. ‘Non-canonical’ Wnt signaling has been only poorly identified. This term usually refers to the pathways activated by Wnt proteins that do not lead to β-catenin stabilization or β-catenin-mediated gene transactivation [16].

The incretin system and Wnt-signaling pathway interact at different stages. Activation of the incretin hormone glucagon-like peptide-1 (GLP-1) signaling pathway by its receptor agonists increases Wnt member 4 (Wnt4) expression and activates the canonical Wnt signaling pathway to promote β-cell proliferation [17–19]. On the other hand, canonical Wnt signaling regulates the expression of GLP-1 in intestinal L cells [20;21]. It has been shown that GLP-1 regulates adipogenesis in 3T3-L1 pre-adipocytes and mesenchymal stem cells originating from human bone marrow (hMSC) [22;23]. To explore the potential mechanism coupling GLP-1 receptor activation to enhanced adipogenesis, we sought a connection between adipogenic markers and Wnt signaling following GLP-1 treatment in 3T3-L1 cells. We show here that the engagement of GLP-1 receptor (GLP-1R) directly regulated the Wnt4-β-catenin pathway during adipocyte differentiation, providing a mechanism for adipose tissue to adapt to metabolic stress.

## Methods and Procedures

### Materials

Cell culture reagents, 5-bromo-2'-deoxyuridine (BrdU), anti-BrdU antibody, DNase I and TRIzol, were purchased from Life Technologies (Carlsbad, CA, USA). GLP-1, Exendin9-39 (Ex9), isobutylmethylxanthine (IBMX), dexamethasone (Dex), 4',6-diamidino-2-phenylindole (DAPI), 3-(4,5-dimethylthiazol-2-yl)-2,5-diphenyltetrazolium bromide (MTT) and Oil Red O were acquired from Sigma Chemical (St. Louis, MO, USA). Complete Protease Inhibitor Cocktail was purchased from Roche Applied Science (Mannheim, Germany). TaKaRa PrimeScript^TM^ RT reagents kits and TaKaRa SYBR premix Ex Taq were acquired from TaKaRa Bio (Kyoto, Japan). Antibodies for β-catenin, (Ser^37^/Thr^41^) non-phospho-β-catenin and FITC-conjugated anti-rabbit and anti-mouse IgG antibodies were purchased from Cell Signaling Transduction (Boston, MA, USA). Antibodies for Wnt4 and GAPDH were acquired from Santa Cruz Biotechnology (Santa Cruz, CA, USA). Lifectamine2000 and siRNA control were purchased from Life Technologies (Grand Island, New York, USA). All other chemicals of analytical grade were acquired from Dingguo Bio (Shanghai, China).

### 3T3-L1 Cell culture and differentiation

3T3-L1 pre-adipocytes were cultured as previously described [24]. Briefly, until confluence, the cells were maintained in high glucose DMEM containing 25 mM glucose, 10% fetal bovine serum and antibiotics, and confluent pre-adipocytes were grown for another 2 days in culture medium supplemented with 1 μM insulin, 0.5 mM IBMX, and 0.1 μM Dex (MDI) and for further 3 days in culture medium with 1 μM insulin. After this period, 3T3-L1 cells were grown for 3–6 more days in culture medium, after which at least 95% of the cells had accumulated fat droplets.

### Assessment of 3T3-L1 lipid accumulation

Lipid accumulation of differentiated 3T3-L1 adipocytes was determined by quantitative Oil Red O staining. Briefly, 14 days after the induction of differentiation, the cells were fixed for 20 min with 4% formaldehyde, followed by incubation with Oil Red O for 30 min. The dye was eluted by the addition of 100 μl (96-well plate) of isopropanol per well and then was measured by reading its absorbance at 540nm.

### RNA extraction and quantitative real-time PCR

Total RNA was extracted from 3T3-L1 adipocytes using TRIzol reagent. After the RNA was treated with DNase I, 1.0 μg of RNA was reverse-transcribed using TaKaRa PrimeScript^TM^ RT reagents kits, according to the manufacturer’s instructions. Quantitative real-time polymerase chain reaction (qPCR) was performed with ABI Prime 7500, using approximately 2–4 μl reverse-transcribed reaction diluted 10 times each. Samples were prepared using TaKaRa SYBR premix Ex Taq, according to the manufacturers’ instructions. After the reaction, each PCR product was verified for its single amplification by melting curve analysis. The gene-specific primers for amplification are listed in Table 1. Gene expression levels were normalized to the expression of GAPDH.

**Table 1:** Forward and reverse primers used for qPCR.

	Forward	Reverse
Wnt4	CTCAAAGGCCTGATCCAGAG	TCACAGCCACACTTCTCCAG
Wnt5a	ACTGGCAGGACTTTCTCAAGGACA	GCCTATTTGCATCACCCTGCCAAA
Wnt5b	CCCCAGGCCAGAGAAAGC	CCTCCCCGATGTAGGACAT
Wnt6	TGTCAGTTCCAGTTCCGTTTCCGA	ACACGAAAGCTGTCTCTCGGATGT
Wnt10b	TGGCTGTAACCACGACATGGACTT	CTGACGTTCCATGGCATTTGCACT
β-Catenin	TGCAGCTTCTGGGTTCCGATGATA	AGATGGCAGGCTCAGTGATGTCTT
PPARG2	TCGCTGATGCACTGCCTATG	GAGAGGTCCACAGAGCTGATT
CEBPA	CAAGAACAGCAACGAGTACCG	GTCACTGGTCAACTCCAGCAC
CEBPB	GGTCGAGCGCAACAACATC	CTCGGGCAGCTGCTTGAACAA
CEBPD	AACCCGCGGCCTTCTACGAG	CACGGCGGCCATGGAGTCAA
LPL	GGGAGTTTGGCTCCAGAGTTT	TGTGTCTTCAGGGGTCCTTAG
GAPDH	TGTGACGTTGACATCCGTAAAGAC	TCCACACAGAGTACTTGCGCTC

### RNAi experiment

Two target regions (1:591–611 and 2:651-671ref) for Wnt4 were selected using the QIANGEN siRNA online design tool. The scrambled fragment 5’-AAGAGGAGCATATTGGGAAGA-3’, which does not have similarity with any mRNA listed in Genebank, was used as a negative control. Transfection of siRNA into 3T3-L1 cells was performed with Lifectamine 2000 according to the manufacturer’s instruction.

### Western blot analysis and subcellular fractionation

Cytoplasmic and nuclear protein fractions were prepared as described previously [25]. Briefly, 10-cm dish-cultured cells were washed with ice-cold phosphate buffered saline (PBS) and were removed from the culture plate by gentle scraping with a cell-scraper in 2 ml of ice-cold fractionation buffer (250 mMsucrose, 20 mM HEPES, pH 7.4, 1 mM EDTA, 1 mM EGTA, 10 mM KCl, 1.5 mM MgCl_2_, 1 mM dithiothreitol and complete protease inhibitor cocktail). The lysate was passed through a 25 G needle 10 times using a 1 ml syringe and was left on ice for 20 min. The nuclear pellet was centrifuged out at 720 g at 4°C for 5 min, and the supernatant was collected for membrane fraction by ultracentrifugation. The nuclear pellet was further washed with fractionation buffer and was passed through a 21 G needle 21 times. Then, the nuclear fraction was collected after centrifugation at 3000 rpm for 5 min at 4°C. For the membrane fraction, the supernatant was centrifuged at 150,000 G for 1 hr. The supernatant was the cytosolic fraction, and the pellet was washed and centrifuged again at 100,000 G for 45 min to obtain the membrane protein pellet. The fractionated proteins were lysed with RIPA buffer (20 mM Tris-HCl pH 7.5, 150 mM NaCl, 1% Nonidet P-40, 0.1% SDS, 2 mM EDTA, 2 mM EGTA, 10% glycerol, 20 μg/ml leupeptin, 20 μg/ml aprotinin, 1 mM phenylmethylsulfonyl fluoride (PMSF), 25 mM β-glycerophosphate, 5 mM sodium orthovanadate, and 5 mM NaF). The cell lysates were resolved by SDS-PAGE as previously described [24]. The proteins were transferred to PVDF membrane (Immun-Blot PVDF membrane; Bio-Rad, Hercules, CA, USA) and immunoblotted with primary antibodies overnight. Specifically bound primary antibodies were detected with horseradish peroxidase (HRP)-coupled secondary antibody and enhanced chemiluminescence.

### Immunostaining of β-catenin

Cells were induced for differentiation for the indicated amount of time as described in the legend, with or without GLP-1. After fixation, the cells were permeabilized and then stained with anti-β-catenin antibody. Finally, FITC-conjugated anti-rabbit IgG antibody was added. Images were obtained using a Nikon fluorescence microscope.

### MTT colorimetric assay

At 60% confluence, 3T3-L1 pre-adipocytes were kept in serum-free DMEM for 6 h and then induced in DMEM with 0.5% BSA medium for proliferation, with or without GLP-1. For MTT assay, the cells were incubated with MTT for 4 h, and DMSO was then added to dissolve the MTT formazan crystals. Absorbance was read at 590 nm.

### BrdU incorporation assay

For Brdu incorporation assay, cells were incubated with 10 μM BrdU for 2 h and fixed with Carnoy's fixative (3 parts methanol to 1 part glacial acetic acid) for another 20 min. Subsequently, the cells were denatured by 2 M HCl and were stained with anti-BrdU monoclonal antibody. FITC-conjugated anti-mouse IgG antibody was added, followed by DAPI counterstaining.

### Statistical analysis

The values are presented as means ±S.E.M.s. Comparisons between groups were performed with Student’s unpaired *t*-test and, in cases of multiple time points and treatments, by one-way ANOVA. P values <0.01 were considered to be highly significant and <0.05 were considered to be significant.

## Results

### 1. GLP-1 enhanced gene expression and cell proliferation in 3T3-L1 pre-adipocytes

**Fig 1:**
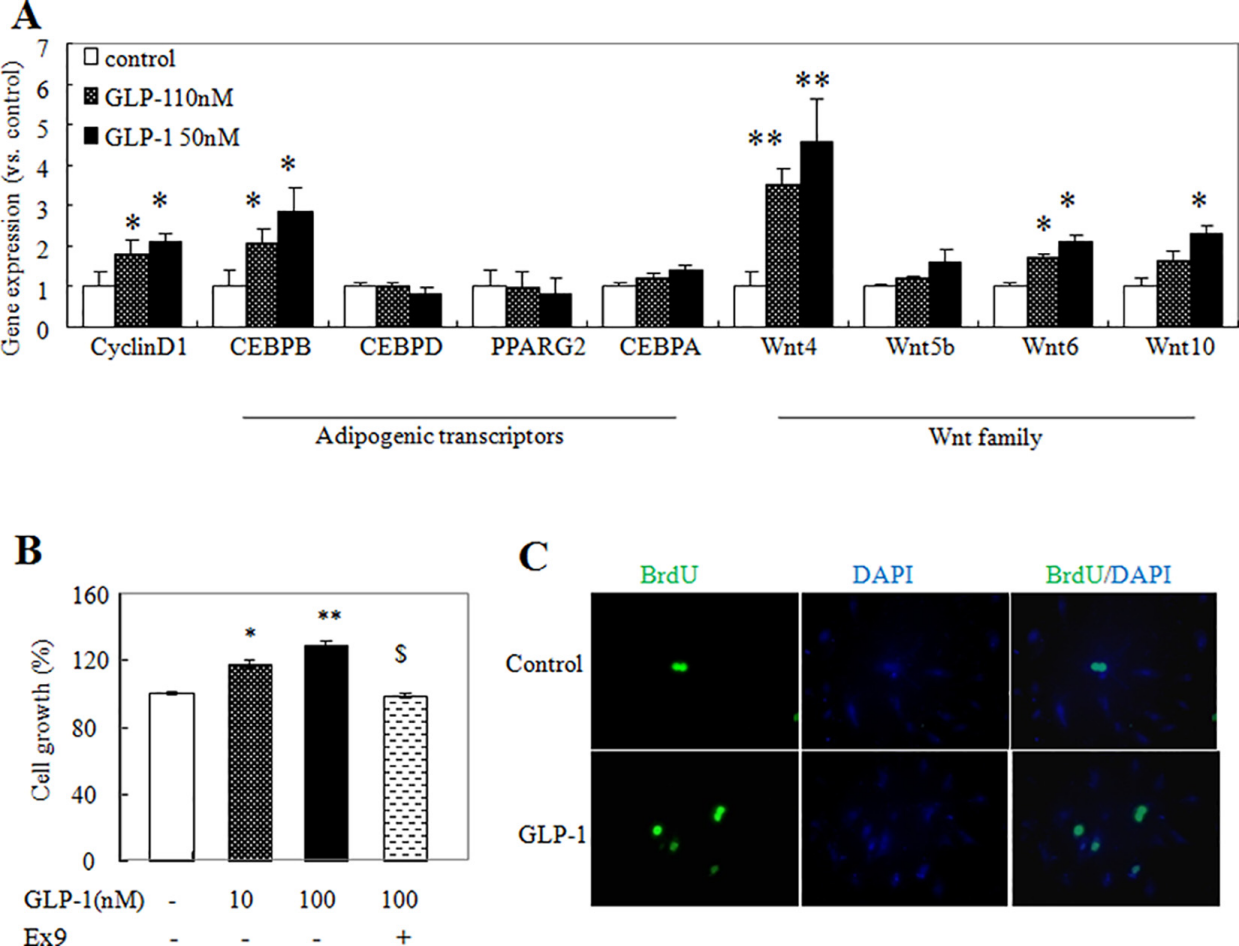
GLP-1 regulated gene expression and proliferation in 3T3-L1 pre-adipocytes. At 60% confluence, 3T3-L1 pre-adipocytes were kept in serum-free DMEM for 6 h and then were treated with GLP-1 for 17 h. (A) Extraction of total RNA and qPCR with gene specific primers were performed. The proliferation of cells was measured by MTT assay (B) and BrdU staining (C). (B) Cells were pretreated with Ex9 for 30 min before GLP-1 was added. (C) FITC-conjugated antibody was added to detect BrdU, and DAPI was added to counterstain nuclei. **P<0.01, *P<0.05, compared with controls. n = 3–6.

We investigated whether GLP-1 regulated Wnt synthesis in vitro. 3T3-L1 pre-adipocytes were cultured with or without GLP-1 (10nM and 50 nM) for 17 h. As demonstrated in Fig 1A, both 10 nM and 50 nM of GLP-1 significantly increased mRNA levels of CEBPB, Wnt4 and Wnt6. The effects of GLP-1 on Wnt10 were found to be dose-related; 50 nM of GLP-1 significantly increased the production of Wnt10, while 10 nM had no significant effect (Fig 1A).These data suggested that GLP-1 would regulate adipocyte differentiation through modulation of the Wnt signaling pathway.

We next examined the effects of GLP-1 on the proliferation of pre-adipocytes. GLP-1 increased the gene expression levels of cyclin D1 at doses of 10 nM and 50 nM (Fig 1A). MTT assay showed that GLP-1 increased cell numbers, which were blocked by pretreatment with a specific GLP-1R antagonist, Ex9 (5 nM)[24] (Fig 1B), thus suggesting a GLP-1R dependent mechanism. Consistently, BrdU staining (Fig 1C) showed that treatment with GLP-1 increased proliferation of the pre-adipocytes. These data suggested that GLP-1-promoted pre-adipocyte growth might contribute in part to mature adipocyte formation.

### 2. GLP-1 promoted adipocyte differentiation in 3T3-L1 cells

**Fig 2:**
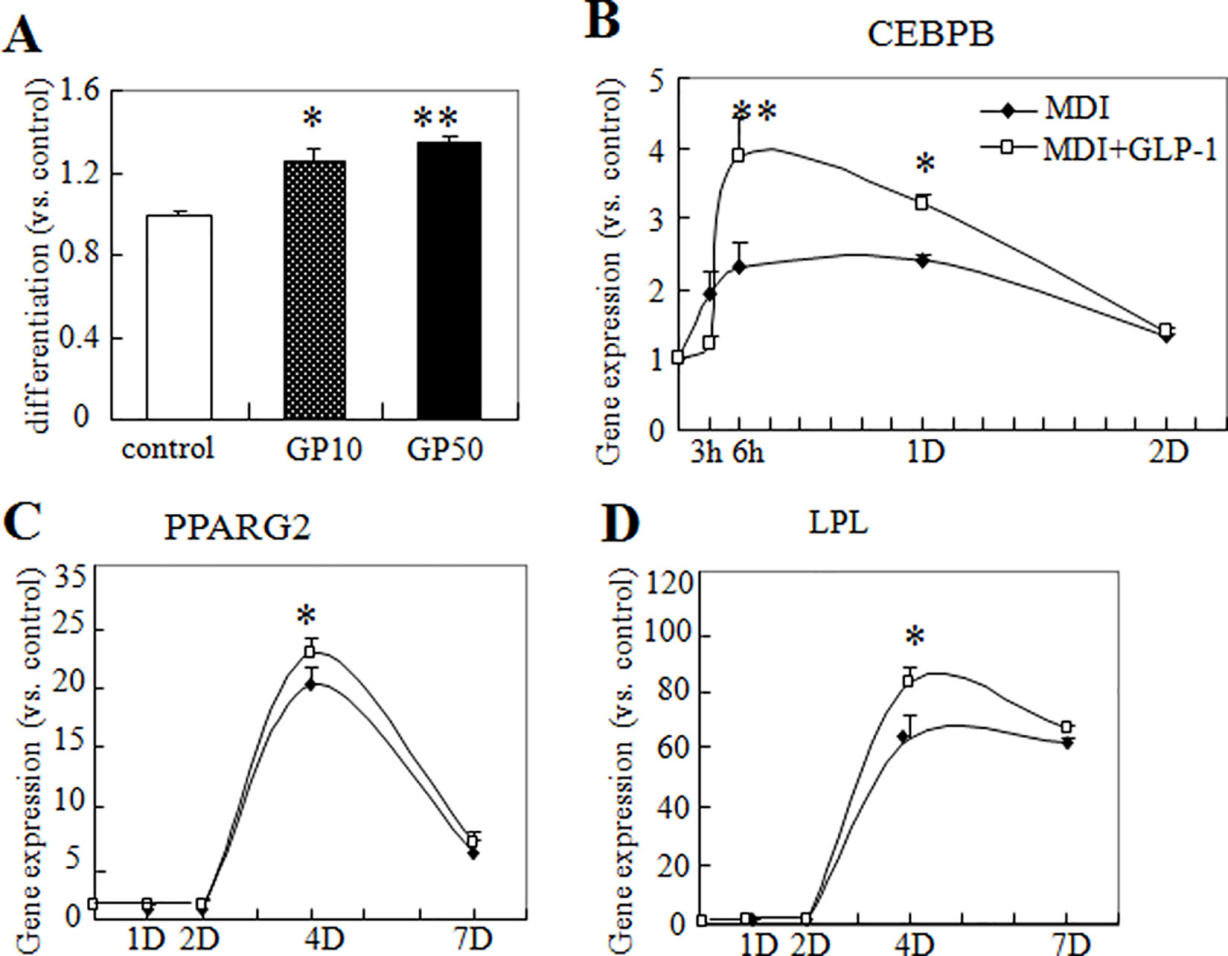
GLP-1 promoted adipogenic differentiation. **3T3-L1 cells were induced to differentiate by MDI with or without GLP-1.** (A) Mature adipocytes were stained with Oil-Red-O, and the color was then eluted in isopropanol and was measured for absorbance at 540 nm. The expression profiles of CEBPB (B), LPL (C) and PPARG (D) were quantified by qPCR with gene-specific primers at the indicated time points after MDI induction. **P<0.01, *P<0.05, compared with controls. n = 4–10.

We found that the lipid accumulation in differentiated adipocytes treated with GLP-1 was increased in a dose-related fashion (Fig 2A). The expression levels of adipogenic transcription factors were determined in these cells undergoing differentiation. The mRNA levels of CEBPB, PPARG and LPL were increased in a time-dependent fashion. After 3 h of incubation with MDI, an increase in CEBPB expression appeared, and maximum mRNA levels were attained before the 1st day after differentiation induction (Fig 2B). The increase in PPARG expression occurred rapidly as well with a peak on the 4th day after induction (Fig 2C). Furthermore, the expression of LPL increased on the 4th day after induction, and high levels persisted throughout the time course studied (Fig 2D). When the 3T3-L1 cells were stimulated to differentiate in the presence of GLP-1, the expressions of CEBPB, PPARG and LPLwere further increased. This increase in expression was significant at the time when expression of all of these factors was at a maximum (Fig 2B–2D).

### 3. GLP-1 promoted adipogenesis by enhancing the dynamic expression of Wnt4 in differentiating 3T3-L1 adipocytes

**Fig 3:**
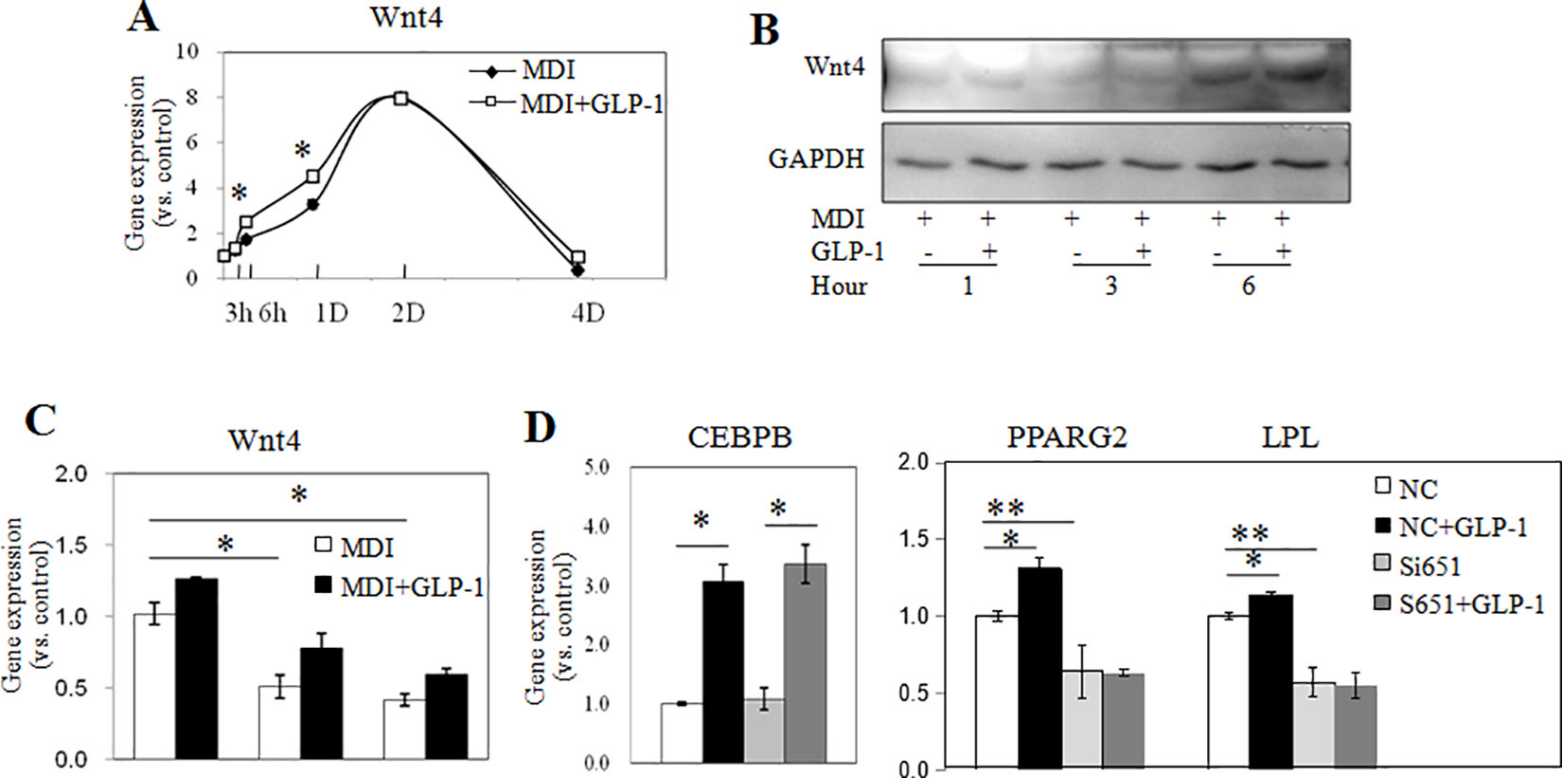
GLP-1 increased the adipocyte differentiation via enhancing Wnt4 expression. 3T3-L1 cells were induced to differentiate by MDI with or without GLP-1. The expression profiles of Wnt4 were quantified by qPCR (A) and western blotting (B) at the indicated time points after MDI induction. Using siRNA651 to silence Wnt4, the expression levels of Wnt4 (C), CEBPB, LPL and PPARG were quantified. NC: negative control. *P<0.05, **P<0.01, compared with controls at the same time point. n = 3–6.

Wnts is reported to be an important regulator of adipogenic differentiation; we investigate whether GLP-1 exerted its action on adipogenesis via regulating the expression of Wnts in pre-adipocytes undergoing differentiation. After 3 h of incubation with MDI, we noticed an increase in Wnt4 expression, with maximum mRNA levels occurring between the 1st day and 4th day after cell induction (Fig 3A). In contrast, the expression levels of Wnt6 and Wnt10 were found to decline rapidly after the initiation of differentiation (see S1 Fig). Interestingly, when 3T3-L1 cells were stimulated to differentiate in the presence of GLP-1, the expression of Wnt4 was found to be further increased statistically significantly at early time points (Fig 3A). GLP-1 did not affect the dynamic expression of Wnt6 and Wnt10 (see S1 Fig). Consistently, the protein production of Wnt4 increased during cell differentiation, and GLP-1 treatment enhanced this increase (Fig 3B).

Wnt4 is known to be an important accelerator of adipogenesis. Silencing Wnt4 via siRNA (Fig 3C) significantly decreased the increment in PPARG and LPL level, while had no obvious effect on CEBPB level (Fig 3D). These results suggest that Wnt4 signaling activated the transcription of PPARG independent of C/EBPB. The effect of GLP-1 was blocked by knocking down Wnt4. Thus, our study showed that GLP-1 promoted the adipogenic differentiation via a novel pathway through Wnt4 mediated transcriptional activation of PPARG.

### 4. GLP-1 promoted adipogenesis through translocation of β-catenin to the plasma membrane

**Fig 4:**
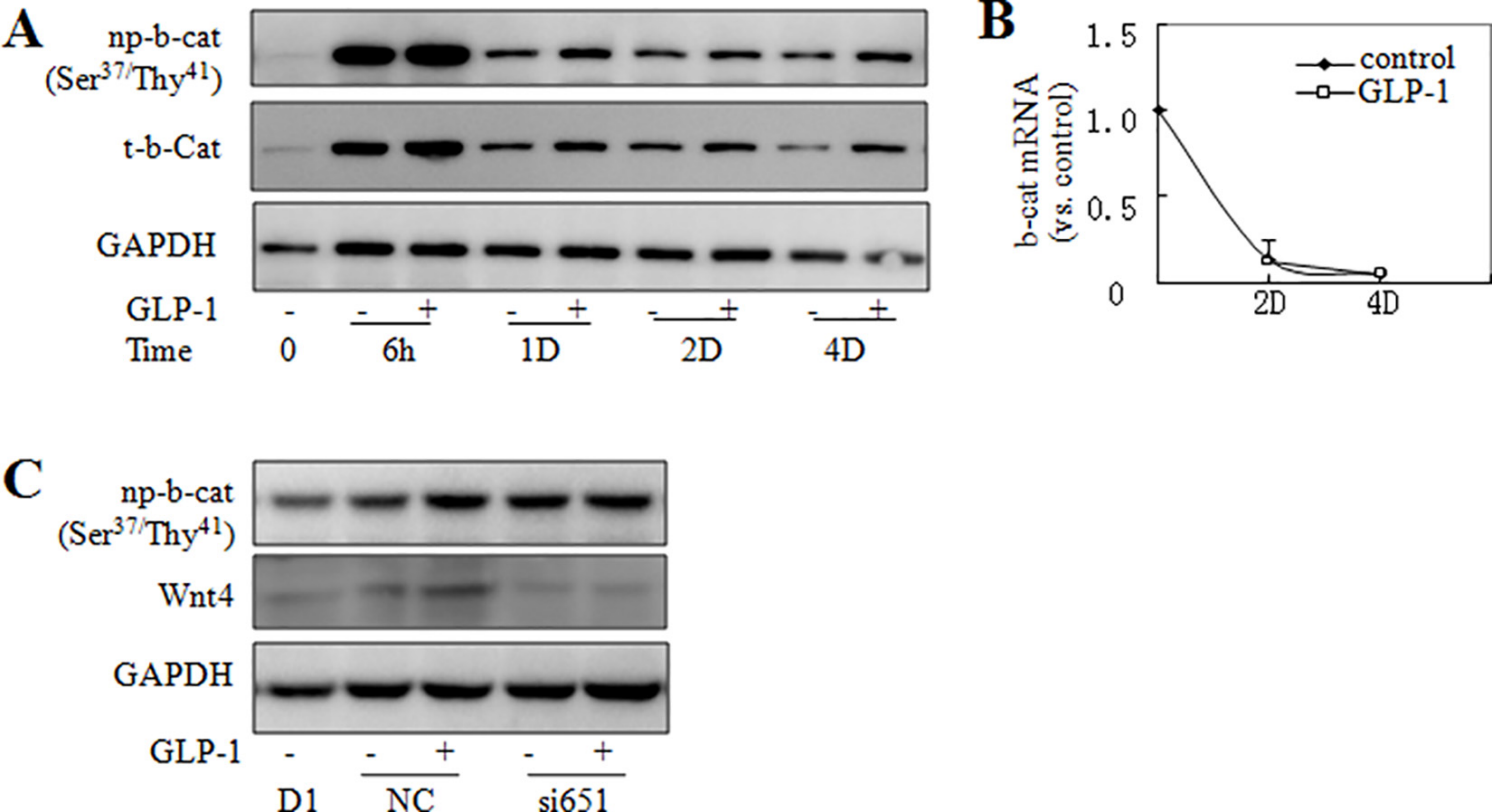
GLP-1 regulated β-catenin via Wnt4 in cells undergoing adipogenesis. 3T3-L1 cells were induced to differentiate by MDI with or without GLP-1. (A) Western blotting was performed to measures the level of total (t-β-cat) and (Ser^37^/Thr^41^) non-phosphorylated β-catenin (np-β-cat) and the expression profile of β-catenin was quantified by qPCR (B) with gene-specific primers at the indicated time points after MDI induction. (C) Using siRNA651 to silence Wnt4, the levels of (t-β-cat) and (Ser^37^/Thr^41^) non-phosphorylated β-catenin (np-β-cat) were detected. NC: negative control.

β-catenin is the major downstream mediator of Wnts. Stimulation of canonical Wnt signaling led to the stabilization of β-catenin through inhibition of its phosphorylation at Ser^37^ and Thr^41^ [26].We tested whether GLP-1 modified the phosphorylation level of β-catenin in cells under differentiation. To accomplish this goal, we used an antibody that only recognizes β-catenin that is not phosphorylated at residues Ser^37^ and Thr^41^ (np-β-catenin). When differentiation was initiated, the level of np-β-catenin protein rapidly increased (at 6h time point) (Fig 4A) and the synthesis of β-catenin declined (afterwards) (Fig 4A) due to transcription inhibition (Fig 4B). GLP-1 treatment had no effect on the transcription and non-phosphorylated proteins level of β-catenin at the early stage of differentiation, while on the 1st day after differentiation the total protein level of β-catenin was modestly increased by GLP-1 treatment. Silencing Wnt4 blocked the increase in β-catenin level by GLP-1 (Fig 4C). These results suggest that GLP-1 might increase the stability of β-catenin via Wnt4.

**Fig 5:**
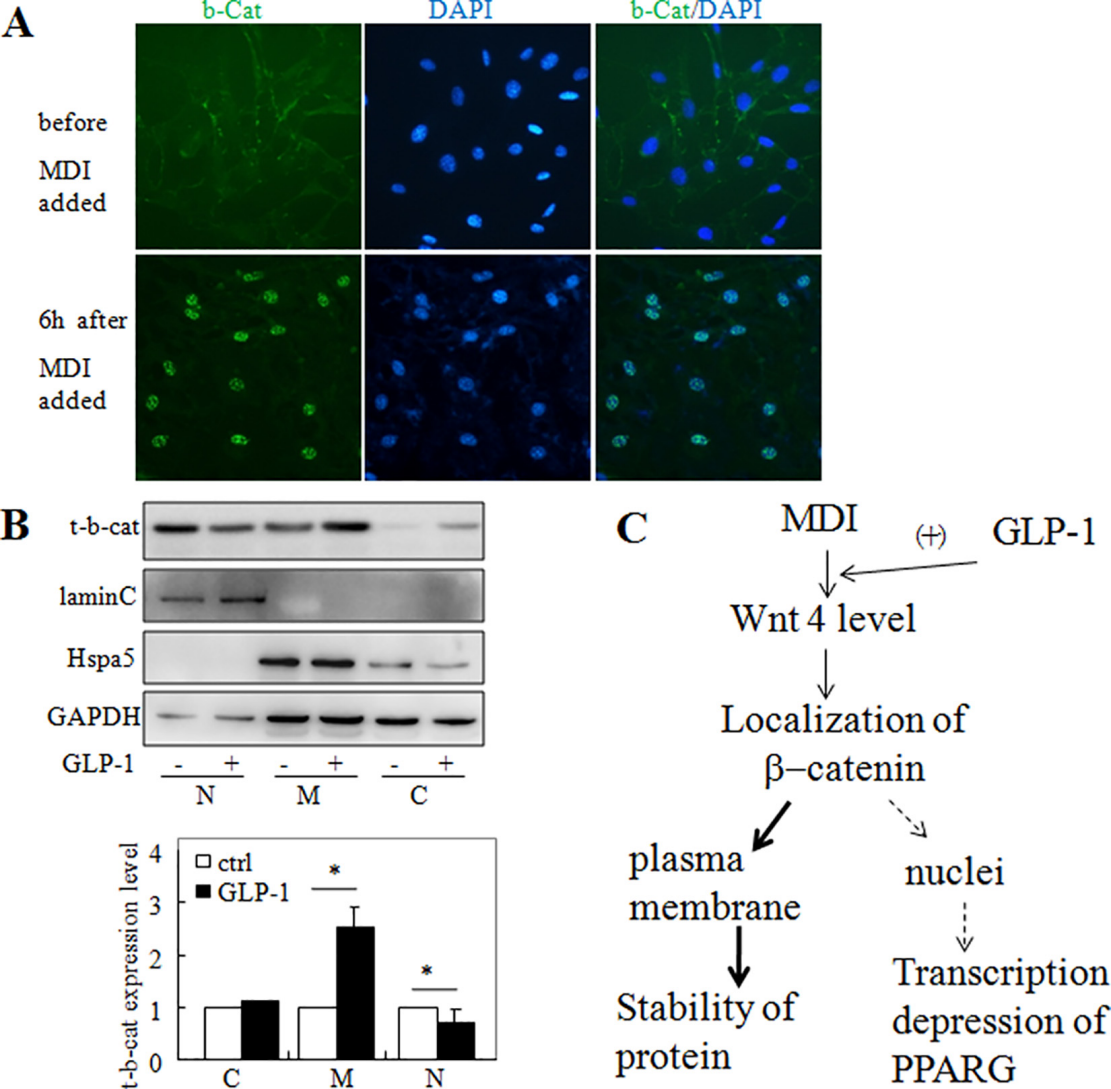
GLP-1 redirected β-catenin to the plasma membrane to promote adipocyte differentiation. (A) Immunofluorescence staining showed nuclear translocation of β-catenin after differentiation began. (B) Subcellular fractionation was performed to qualify the subcellular distribution of β-catenin at 6 h after MDI induction. The purity of the fractions was estimated by measuring the amounts of Hspa5 in the membrane fraction, Lamin C in the nucleus, and GAPDH in the cytosol (upper). Densitometric analysis corresponded to three independent experiments (lower). *P<0.05. n = 3–6. (C) Model depicting the proposed mechanism of Wnt4-dependent GLP-1 action on β-catenin translocation. Abbreviation: MDI: culture media with IBMX, Dex and insulin.

β-catenin is reported to be a transcription repressor during adipogenic differentiation. Wnt4 is able to antagonize canonical Wnt signaling by specifically redirecting β-catenin to the plasma membrane [27]. We tested the subcellular localization of β-catenin in cells undergoing differentiation. Initiation of differentiation induced a quick translocation of β-catenin from cytoplasm to nucleus by immunofluorescence staining (Fig 5A). At 6 h after MDI induction, the accumulation of β-catenin in the nucleus reached the maximum together with morphological change of cells. Thus, it is much likely that the negative regulation mechanism mediated by β-catenin was quickly and transiently initiated at the very early stage of the differentiation. Furthermore, cytoplasmic, plasma membrane and nuclear fraction extracts was prepared from the differentiating cells with or without GLP-1 treatment (Fig 5B).The purity of the fractions was estimated by measuring the amounts of Hspa5 (heat-shock 70 kDa protein 5) in the membrane fraction, lamin C in the nucleus, and GAPDH in the cytosol. Membrane-bound β-catenin was significantly increased in the GLP-1-treated cells, compared with the control cells (middle panel, membrane, and the graphical representation), while a modest but significant decrease in β-catenin in the nuclei was observed in cells treated with GLP-1 (left panel, nucleus, and the graphical representation). These results suggested that GLP-1 might prevent the transcriptional depression effect of β-catenin by directing it to membrane, thereby resulting in facilitating adipocyte differentiation.

Translocation to membrane by Wnt4 avoids β-catenin to be degradated in the cytoplasm [27], which might explain the β-catenin level increase at 1 day after differentiation by GLP-1 treatment (Fig 1A). These results suggested that the action of GLP-1 on Wnt4 specifically triggered β-catenin relocalization to the plasma membrane in cells undergoing adipogenic differentiation, thereby preventing its involvement in gene transcriptional regulationin the nucleus.

## Discussion

GLP-1 exerts a variety of biological functions, including enhancing glucose-dependent insulin secretion, suppressing glucagon secretion, slowing gastric emptying, reducing food intake and stimulating satiety [28]. Using a gene therapy strategy, we previously found that high-fat diet-fed mice had decreased body weight gains, improved circulating lipidemia and greater insulin sensitivity following GLP-1 treatment [29].These findings inspired us to explore the local effects of GLP-1 on adipose tissues. The 3T3-L1 cell line is a widely used model for adipocyte formation and function [30]. GLP-1R was cloned in 3T3-L1 preadipocytes and adipocytes, and its activation stimulated the synthesis of visfatin [24]. Here, we demonstrated that GLP-1 promoted the differentiation of adipocyte in 3T3-L1 cells by increasing the expression of adipogenic transcription factors. Our data were consistent with previous findings by Challa ID et al [22].

Suppression of adipogenesis has been linked to increased insulin resistance [31], while enhancement of adipogenesis resulted in increased glucose disposal and high adiponectin secretion [32]. In addition, during positive caloric balance, increased storage of energy occurs through adipogenesis from pre-adipocytes. Thus, it is likely that, in response to food intake, secreted GLP-1 induces adipocyte precursors to differentiate into mature adipocytes. This process could benefit whole body insulin sensitivity by decelerating ectopic lipid accumulation and decreasing weight gain.Our data suggested that GLP-1 increased the adipogenesis of 3T3-L1 cells via multiple pathways, including by promoting the proliferation of pre-adipocytes (Fig 1B), enhancing CEBPB expression (Fig 2B) and blocking β-catenin action in differentiating cells (Fig 4). Activation of CEBPA expression by CEBPB during adipogenesis requires a PPARG -associated repression of HDAC1 at the CEBPA gene promoter, though they mutually promote the expression of each other thereafter [33]. PPARG and CEBPA have been reported to cooperate in activation of a few adipocyte genes with different patterns of synergy, whereas the expression of LPL mRNA could be induced by both PPARG and CEBPA [34]. In our study, GLP-1 enhanced the expression of PPARG while had no obvious effect on the expression of CEBPA, and then increased the transcription of LPL.

Wnt signaling possibly serves to regulate adipose expansiontightly, to meet energy storage demands [11]. The finding that disruption of Wnt/β-catenin signaling led to spontaneous adipogenesis indicated that endogenous Wnts restrained preadipocytes differentiation [35]. One likely candidate for such an anti-adipogenic WNT signal is Wnt10b. In 3T3-L1 preadipocytes, overexpression of Wnt10b stabilized β-catenin and blocked adipogenesis, while addition of Wnt10b anti-sera to culture media promoted adipocyte differentiation [36]. Another candidate is Wnt6. Overexpression of Wnt6 stabilized β-catenin and prevented adipogenesis [35]. MSCs give rise to numerous cell types, including adipocytes and osteoblasts. Wnt/β-catenin signaling is an important regulator of the fate of MSCs. Wnt6 was a more potent endogenous regulator of MSC fate thanWnt10a or Wnt10b, at least in vitro[35]. Compared to 3T3-L1 cells, adipogenic differentiation of hMSCs was inhibited by GLP-1. Our data showed that GLP-1 had the ability to regulate the expression of both Wnt6 and Wnt10. Thus, we propose that the regulation pattern of GLP-1 on Wnts in different cells might lead to the decrease in adipocyte formation from hMSCs.

The phosphorylation level of β-catenin is tightly regulated by Wnt signaling. When adipogenesis was initiated, the expression levels of Wnt6 and Wnt10b rapidly and sharply decreased. In addition, expression levels of the Wnt receptors Fz1, Fz2 and Fz5 also decreased upon the induction of differentiation [13]. Thus, β-catenin should be phosphorylated and degraded in the cytoplasm. However, we found rapid dephosphorylation and translocation of β-catenin to the nucleus when differentiation was initiated. It was previously showed that ectopically expressed, constitutively active chimeras between Wnt8 and Fz1 or overexpression of Wnt10b in pre-adipocytes and MSCs increased the levels of β-catenin and inhibited adipogenesis by blocking the induction of the key transcription factors PPARG and CEBPA [37]. Therefore, it is possible that, when differentiation was initiated, β-catenin translocated to the nucleus to antagonize CEBPB and CEBPD by inhibiting the transcription activity of PPARG and CEBPA.

Wnt4 is essential for cell proliferation and development in a broad range of tissues [38;39]. In particular, Wnt4 was abundantly expressed in pancreatic β-cells and acted as regulator of β-cell proliferation and TNF-α release [17]. The GLP-1 analogue Ex4 dose-dependently increased the expression of Wnt4 in the mRNA and the protein levels in pancreatic β-cells, and the GLP-1 antagonist Ex9 abolished these effects. While knocking down Wnt4 decreased the β cell proliferation to 45% of the controls, it was suggested that GLP-1 promoted β-cell proliferation in part via the Wnt4 pathway [17]. In adipocytes, inhibition of Wnt4 expression prevented the cytoplasmic accumulation of triacylglycerol and decreased the expression of adipogenesis-related genes [40]. Conversely, overexpression of Wnt4 reduced the transcription activity of β-catenin by translocation to the plasma membrane [41]. Our present study showed that, when adipogenesis was initiated, the expression level of Wnt4 changed dramatically and was closely related to adipocyte differentiation [40]. During this time frame, GLP-1 increased the level of membrane-bound β-catenin. Localization at the plasma membrane could help β-catenin to avoid degradation by phosphorylation in the cytoplasm. In fact, in the early stage of differentiation, when β-catenin expression decreased, and Wnt4 expression was at its peak time point, the total β-catenin level was higher in GLP-1-treated cells. Later, when Wnt4 expression decreased, GLP-1 lost its action on β-catenin. Thus, our data suggested that Wnt4 was an important mediator of the effects of GLP-1 on the activity and stability of β-catenin during adipogenic differentiation. A model for the Wnt4/β-catenin-dependent action of GLP-1 during adipocyte differentiation is shown in Fig 5.

In conclusion, our data demonstrated that GLP-1 promoted adipogenesis, in part by enhancing Wnt4 expression. On the molecular level, we revealed a novel pathway of β-catenin based on its translocation and accumulation in the nucleus during the early stages of differentiation. GLP-1 redirected β-catenin to the plasma membrane via the Wnt4 pathway. Therefore, GLP-1 could exert part of its effects on adipocytes by upregulation of Wnt4, modulating β-catenin signaling in pre-adipocytes undergoing differentiation.

## Supporting Information

S1 Fig The effect of GLP-1 on the expression profiles of Wnt6 and Wnt10. The transcription level of Wnt6 and Wnt10 were quantified by qPCR at the indicated time points after MDI induction. n = 3–6.
(TIF)

## Abbreviations

GLP-1: glucagon-like peptide-1
GLP-1R: GLP-1 receptor
CEBPA/B/D: CCAAT/enhancer-binding protein-α,-β and –δ
Wnts: wingless-type MMTV integration site family members
Wnt4: Wnt member 4
np-β-catenin: non-phospho-β-catenin at residues Ser^37^ and Thr^41^
GSK3β: glycogen synthase kinase-3β
