# Supplementary figures and images for: Glucagon Like Peptide-1 Promotes Adipocyte Differentiation via the Wnt4 Mediated Sequestering of Beta-Catenin
Source: PLoS One. 2016 Aug 9;11(8):e0160212. doi: 10.1371/journal.pone.0160212 (PMC4978386; doi:10.1371/journal.pone.0160212)

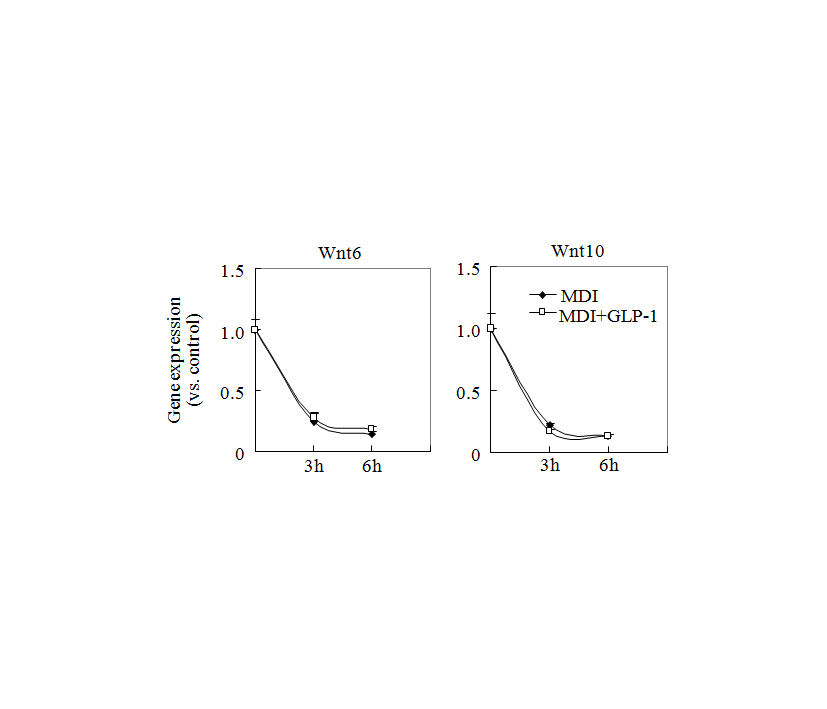

Supplement: S1 Fig — The transcription level of Wnt6 and Wnt10 were quantified by qPCR at the indicated time points after MDI induction. n = 3–6. (TIF) [file pone.0160212.s001.tif]
